# A Cellular Mechanism Underlying Enhanced Capability for Complex Olfactory Discrimination Learning

**DOI:** 10.1523/ENEURO.0198-18.2019

**Published:** 2019-02-12

**Authors:** Naveen Chandra, Richa Awasthi, Togba Ozdogan, Friedrich W. Johenning, Barbara Imbrosci, Genela Morris, Dietmar Schmitz, Edi Barkai

**Affiliations:** University of Haifa, Haifa 3498838, Israel

**Keywords:** GlUk2 receptors, intracellular recordings, meta-learning, olfactory-learning, pyramidal neurons, spost-burst AHP

## Abstract

The biological mechanisms underlying complex forms of learning requiring the understanding of rules based on previous experience are not yet known. Previous studies have raised the intriguing possibility that improvement in complex learning tasks requires the long-term modulation of intrinsic neuronal excitability, induced by reducing the conductance of the slow calcium-dependent potassium current (sI_AHP_) simultaneously in most neurons in the relevant neuronal networks in several key brain areas. Such sI_AHP_ reduction is expressed in attenuation of the postburst afterhyperpolarization (AHP) potential, and thus in enhanced repetitive action potential firing. Using complex olfactory discrimination (OD) learning as a model for complex learning, we show that brief activation of the GluK2 subtype glutamate receptor results in long-lasting enhancement of neuronal excitability in neurons from controls, but not from trained rats. Such an effect can be obtained by a brief tetanic synaptic stimulation or by direct application of kainate, both of which reduce the postburst AHP in pyramidal neurons. Induction of long-lasting enhancement of neuronal excitability is mediated via a metabotropic process that requires PKC and ERK activation. Intrinsic neuronal excitability cannot be modulated by synaptic activation in neurons from GluK2 knock-out mice. Accordingly, these mice are incapable of learning the complex OD task. Moreover, viral-induced overexpression of Gluk2 in piriform cortex pyramidal neurons results in remarkable enhancement of complex OD learning. Thus, signaling via kainate receptors has a central functional role in higher cognitive abilities.

## Significance Statement

In this study, we describe the molecular and cellular mechanism for complex learning. Previous studies suggest that complex learning is accompanied by enhanced intrinsic neuronal excitability, induced by reducing the conductance of a slow potassium current in most neurons in the relevant brain areas. Using complex olfactory discrimination learning, we show that the kainate GluK2 subtype glutamate receptor is necessary and sufficient for complex learning. Intrinsic neuronal excitability cannot be modulated in neurons from GluK2 knock-out mice. Accordingly, these mice are incapable of learning the complex task. Moreover, viral-induced overexpression of Gluk2 in piriform cortex pyramidal neurons enhances complex learning remarkably. Thus, postsynaptic activation of GluK2 receptors has a central functional role in higher cognitive abilities.

## Introduction

Training rodents in a complex olfactory discrimination (OD) task results in enhanced performance over repeated training sessions, as they need to understand and apply a set of complex rules (Saar and Barkai 2003; Zelcer et al., 2006; Chandra and Barkai, 2018). Since such high-order and complex learning would presumably require the active participation and modulation of multiple brain areas and neuronal networks, it was suggested to be mediated by the combined action of large groups of neurons (Hasselmo and Stern, 2018). At the cellular and synaptic level, the simultaneous modulation of many synaptic connections and neurons has been suggested to occur via a process termed metaplasticity. Metaplasticity is defined as a persistent and activity-dependent change in the functional state of the neuron that affects future synaptic plasticity (Abraham and Bear, 1996; Abraham, 2008; Hulme et al., 2013). Notably, it is yet to be shown that metaplasticity mechanisms that could prepare neuronal networks to encode specific content indeed underlie complex learning on the behavioral level (Hulme et al., 2013).

In this context, learning induced enhancement in intrinsic neuronal excitability is a global phenomenon observed in a multitude of brain regions and behavioral tasks (Moyer et al., 1996; Thompson et al., 1996; Saar et al., 2001; Oh et al., 2003; Zelcer et al., 2006; Motanis et al., 2014). In particular, for the olfactory discrimination learning paradigm central to this study, global changes in neuronal intrinsic excitability occur in different brain regions, specifically the piriform cortex (Saar et al., 1998, 2001), the hippocampal CA1 area (Zelcer et al., 2006), and the basolateral amygdala (Motanis et al., 2014).

Spike frequency adaptation is an important regulatory mechanism for intrinsic excitability. In hippocampal and cortical pyramidal neurons, spike frequency adaptation is modulated by medium and slow afterhyperpolarizations (AHPs), generated by calcium-dependent hyperpolarizing potassium currents (Madison and Nicoll, 1984; Constanti and Sim, 1987). Indeed, learning-induced long-term reduction in the postburst AHP and the resulting enhanced excitability is mediated by a long-term decrease in the conductance of the slow calcium-dependent potassium current (sI_AHP_), in both the piriform cortex and hippocampus (Power et al., 2002; Brosh et al., 2006). At the molecular level, learning-induced long-term AHP reduction depends on persistent activation of PKC and ERK second-messenger systems (Cohen-Matsliah et al., 2007). It is as yet unclear how AHP-mediated intrinsic excitability modifications are induced during learning.

Within the hippocampal formation, the GluK2 subtype glutamate receptor enhances intrinsic neuronal excitability via noncanonical signaling (Fisahn et al., 2005; Melyan and Wheal, 2011, Chamberlain et al., 2013). Such synaptic activity-induced and kainate-induced AHP reduction is also dependent on PKC and ERK activation (Melyan et al., 2002, 2004; Grabauskas et al., 2007). This noncanonical GluK2 function is distinct from its direct ionotropic action on synaptic transmission (Ruiz et al., 2005). Interestingly, humans lacking the gene for GluK2 suffer from intellectual disability (Motazacker et al., 2007). To date, the noncanonical signaling of GluK2 receptors has been related to neuronal plasticity in terms of long-term potentiation (LTP) only (Petrovic et al., 2017).

Here, we show that signaling via the GluK2 subtype receptor has a key role in complex OD learning. *In vitro* induced long-term AHP reduction is occluded by prior learning-induced AHP reduction. Moreover, GluK2 activity is both necessary and sufficient for the enhancement of complex learning capabilities. Our data suggest that this glutamatergic receptor and the downstream metaplastic AHP reduction have a central role in complex learning.

## Materials and Methods

### Animal training

#### Rat training in complex olfactory learning

*Subjects and apparatus*. Age-matched young adult Sprague Dawley male rats were maintained on a 23.5 h water-deprivation schedule before training, with food available *ad libitum*. Olfactory discrimination training protocol was performed daily on each trained and pseudotrained rat in a four-arm radial maze, as previously described (Saar et al., 1998, 2001), with commercial synthetic odors that are regularly used in the cosmetics and food industry purchased from Value Fragrances & Flavors. Odors were diluted by factor of 1000 before use in the olfactory maze. Odors were composed from natural and naturally derived materials in their formulations. Odors used in this study were as follows: apple, skin musk, and lavender.

*Training*. Olfactory training consisted of 20 trials per day for each rat, as previously described (Saar et al., 1998). In short, in each trial the rat had to choose between two odors (positive cue and negative cue) presented simultaneously. Rats designated to the trained group were rewarded on choosing the positive cue. Rats in the pseudotrained group were rewarded in a random fashion on choosing any odor. The criterion for learning was at least 80% positive-cue choices in the last 10 trials of a training day, as was previously used (Saar et al., 1998; 2001). Rats in the naive group were water restricted, but not exposed to the maze. Typically, two to three trained rats and two to three pseudotrained rats were trained at the same training period. As previously described (Saar et al., 1998, 2001), normal rats required 7–8 d of training for achieving the criterion for learning.

#### Mice training in the complex olfactory learning

*Subjects and apparatus*. GluK2^−/−^ mice on a C57BL/6J background and their wild-type littermates were trained in an olfactory maze similar to that used for rats, with few adjustments to better fit mice. The arms of the maze were enclosed by 20-cm-high walls, and each running track was 10 cm in width and 35 cm in length. Entries to arms to were blocked by doors, which opened at the beginning of each trial. The center area of the maze contained four odor pokes in front of each track. The end of each track contained a drinking well (1.5 cm radius), which received water by means of an automated valve. The maze was controlled by a computer, which sent transistor-transistor logic pulses at the beginning of each trial to cue the active odor pokes, to open the valves to release odors into the pokes, and to activate the valve that pumps water into the drinking wells.

*Training*. Before the start of experiments, each animal was handled for 5 d and was habituated to the training arena for 3 d. The water restriction protocol started 23 h before the first training day. Animals were allowed to drink water daily *ad libitum* in their home cage for 1 h and 15 min after the training.

Each training session consisted of 20 trials. At the beginning of each trial, two odor pokes were illuminated to cue the odor pokes that were active for that trial, and two different odors were presented (varied across different batches of mice), one of which was associated with water reward. If the animal chose to enter the correct track, it received 0.2 ml of water at the end of the track. In case of wrong entry, the animal did not receive any water and walked back to the center of the maze and waited for the next trial to start. Experiments aimed to examine the effect of GluK2 overexpression on complex olfactory learning were performed blind.

#### Simple olfactory task (cookie test)

After the completion of training in the complex olfactory plus maze, the mice were tested for basic olfactory function using the buried food or cookie test. Mice were habituated to butter cookies for 2 d and were subsequently slightly food restricted for 8 h before the cookie test. Each mouse started the test in a 20 × 40 cm cage (identical in shape and size to their home cage) and freely foraged for the cookie, which was buried in the cage bedding. We used latency to find the cookie as the parameter for the cookie test learning.

### Electrophysiology

#### Sharp electrode recordings

**Figure 1.**
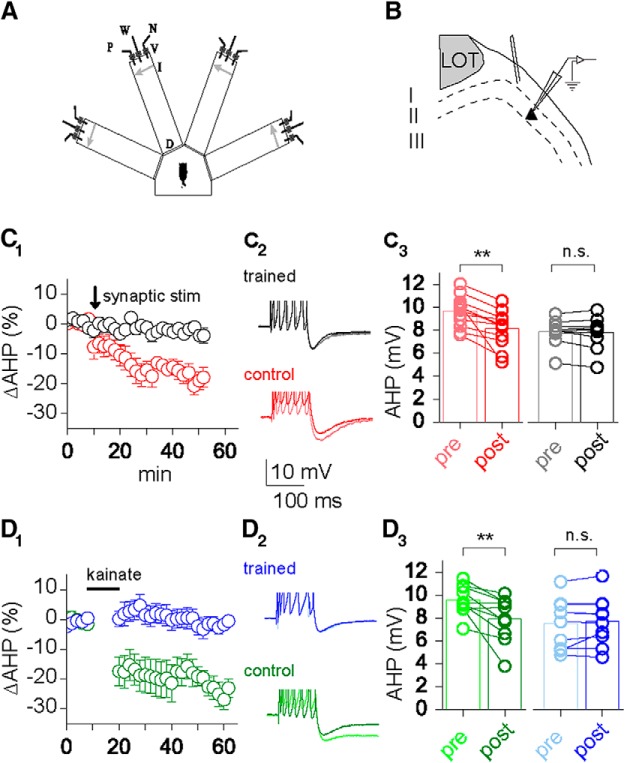
Learning occludes repetitive synaptic stimulation-induced and kainate-induced AHP reduction. ***A***, Schematic description of the four-arm maze. Protocols for trained and pseudotrained rats are similar: an electronic “start” command randomly opens two of eight valves (V), releasing a positive-cue odor (P) into one of the arms and a negative-cue odor (N) into another. Eight seconds later, the two corresponding guillotine doors (D) are lifted to allow the rat to enter the selected arms. On reaching the far end of an arm (90 cm long), the rat body interrupts an infrared beam (I; arrow) and a drop of drinking water is released from a water hose (W) into a small drinking well (trained rats: only if the arm contains the positive-cue odor, pseudotrained rats: random assignment of odors to rewards). ***B***, Position of the intracellular recording electrode in layer II of the anterior piriform cortex in acute coronal brain slices and the stimulation electrode in layer Ib. ***C_1_***, Time line of long-term AHP reduction induced by tetanic synaptic stimulation of trained (black) and control (red) rats. ***C_2_***, Averaged typical traces of a neuron from a trained (black) and pseudotrained (red) rat before (light shade) and after (dark shade) synaptic stimulation. The AP peaks are clipped to facilitate comparison of the postburst AHP. ***C_3_***, Direct comparison of the absolute AHP amplitudes before and 20 min after synaptic stimulation for control (red) and trained (black) rats. ***D_1_***, Time line of long-term AHP reduction induced by kainate application to brain slices of trained (blue) and control (green) rats. ***D_2_***, Averaged typical traces of a neuron from a trained (blue) and pseudotrained (green) rat before (light shade) and after (dark shade) kainate application. The AP peaks are clipped to facilitate comparison of the postburst AHP. ***D_3_***, Direct comparison of the absolute AHP amplitudes before and 20 min after kainate application for pseudotrained (green) and trained (blue) rats. Values are represented as the mean ± SEM. ^**^p < 0.01.

The 400 μm coronal piriform cortex rat brain slices were cut as previously described (Saar et al., 1998) and were kept in oxygenated (95% O_2_ + 5% CO_2_) normal saline Ringer’s solution as follows (in mm): NaCl 124, KCl 3, MgSO_4_ 2, NaH_2_PO_4_ 1.25, NaHCO_3_ 26, CaCl_2_ 2, and glucose 10. Intracellular recordings were obtained from pyramidal cells in layer II of the piriform cortex, with 4 m K-acetate-filled sharp glass microelectrodes at 35^º^C. Several piriform cortex slices were obtained from each rat. Slices were placed in a recording chamber and perfused with Ringer’s solution. Intracellular recordings with sharp electrodes were obtained as previously described (Cohen-Matsliah et al., 2007). Recordings were performed using Axopatch 1D (Molecular Devices), and the data were acquired using pClamp9 (Molecular Devices). All experiments were performed blind; the identity of the rat from which neurons were recorded (naive, trained, or pseudotrained) was not known to the person conducting the experiments and measurements. One to three neurons were recorded from each rat. AHPs were recorded within minutes after good recording conditions were established [resting potential of at least −65 mV and action potential (AP) amplitude of ≥80 mV]. To standardize AHP recordings, neurons were depolarized to holding potential of −60 mV by direct current application via the recording electrode. Postburst AHP amplitude was then measured following a 100 ms depolarizing current step with an intensity that generates six action potentials (Fig. 1*C2* ,*D2*). The AHP amplitude was evoked in responses to intrinsic stimuli applied once every 10 s. The postburst AHP amplitude was measured from baseline to the peak of the hyperpolarizing voltage deflection that follows an evoked train of six action potentials. The stimulus intensity required to evoke six action potentials always remained constant throughout the recording period of each neuron, before and after drug application. Such stability of the required stimulus intensity well coincides with the stability of basic membrane properties, such as the input resistance or resting potential of the neurons, which were not affected by the drugs. Our previous study showed that the AHP amplitudes are not modified in neurons from pseudotrained rats, compared with neurons from naive rats (Saar et al., 1998; Brosh et al., 2006; Cohen-Matsliah et al., 2007; Saar et al., 2012). Therefore, neurons from naive and pseudotrained rats were pooled to form the control group. Synaptic stimulation was delivered via a bipolar tungsten electrode, positioned at layer Ib, to activate the intracortical axons, which interconnect the recorded pyramidal neurons (Fig. 1*B*; Saar et al., 2002) Stimulus intensity was adjusted to evoke PSPs with amplitudes of 10 mV at a membrane potential of −80 mV. Activity-induced AHP reduction was induced by applying 20 repetitive stimuli at 50 Hz.

Drugs were applied into the perfusing Ringer’s solution at the following concentrations: kainite, 200 nm; ERK inhibitor UO126, 30 µm; PKC activator 1-oleoyl-20acety-*sn*-glycerol (OAG), 10 µm. Slices were exposed to drugs for at least 20 min before the recordings. All cells were recorded before and after drug application.

#### Whole-cell patch-clamp recordings

Male C57BL/6 mice, 4–6 weeks of age, were decapitated following isoflurane anesthesia. Brains were transferred to ice-cold artificial CSF (ACSF) slicing solution containing the following (in mm): 87 NaCl, 50 sucrose, 26 NaHCO_3_, 10 glucose, 2.5 KCl, 1.25 NaH_2_PO_4,_ 3 MgCl_2_, and 0.5 CaCl_2_, pH 7.4. Coronal slices from the piriform cortex (400 μm thickness) were cut on a slicer (model VT1200S, Leica) and stored at 32–34°C in the same ACSF slicing solution for 30 min. Subsequently, slices were transferred in a standard ASCF containing (in mm) 119 NaCl, 26 NaHCO_3_, 10 glucose, 2.5 KCl, 1.0 NaH_2_PO_4_, 1.3 MgCl_2_, and 2.5 CaCl_2_, and were allowed to recover for an additional 30 min at room temperature. The ACSF was equilibrated with carbogen (95% O_2_, 5% CO_2_). Recordings were performed in the standard ACSF at 31–32°C in a submerged-type recording chamber perfused at a high rate (5–6 ml/min). Data were collected using a Multiclamp 700A amplifier (Molecular Devices) and sampled at 20 KHz. Whole-cell recordings were performed from layer II pyramidal cells with borosilicate glass electrodes (2–5 MΩ) filled with the following (in mm): 120 K-gluconate, 10 HEPES, 10 KCl, 3 Mg-ATP, 5 EGTA, 2 MgSO_4_, 0.3 Na-GTP, and 14 phosphocreatine. The pH was adjusted to 7.4 with KOH. Neurons were recorded in voltage-clamp mode at −70 mV in the presence of the NMDA receptor blocker, d-AP5 (50 μm) and the GABA_A_ receptor blocker SR-95531 (1 μm). A single extracellular stimulation pulse was delivered every 15 s to evoke postsynaptic currents in the recorded neurons. After achieving a stable baseline, the selective AMPA receptor blocker GYKI-53655 (100 μm) was applied to investigate the potential presence of kainate receptor-mediated currents. In addition, to exclude the presence of extrasynaptic kainate receptors, a train of five stimuli at 200 Hz was delivered under the same recording conditions.

### Statistical analysis

For behavioral experiments, *t* tests were used for statistical comparison between two groups. The effect of repetitive stimulation and drug application was examined for each neuron with and without the treatment, using a paired *t* test. Values throughout the text and in graphs are presented as the mean ± SE.

### Viral injections

#### Apparatus and surgery

Animals were blurred with isoflurane and then anesthetized by the injection of ketamine 10% (0.09 cc/100 g) and Dormitor (0.05 cc/100 g). Before the surgery, rats received treatment with an antibiotic (amoxicillin veterinary, 0.2 ml) and a painkiller (Calmagin, 0.03 ml/100 g). Additional anesthesia (1% isoflurane in O_2_, by inhalation) was supplied as needed. Aseptic surgeries were performed on a commercial stereotaxic device (David Kopf), using a surgical microscope (Zeiss). Physiologic vital conditions were monitored throughout the surgery, and postoperative care was provided to minimize pain and ensure a healthy recovery. Small holes were drilled in the skull to allow the insertion of the syringe in the brain. The locations of the holes were determined according to atlas with bregma as the reference point. A quartz micropipette filled with 200 nl of high-titer lentivirus stock was directed to layer II of the anterior piriform cortex. Virus was pressure injected using a Picospritzer (General Valve). The virus was injected bilaterally. The coordinates in the pirform cortex area were as follows: anteroposterior, +1.3; ML, ±3.5; DV, −8.

The surgery took place in a lentivirus special room. To ensure virus expression and to allow rats to recover from the surgery, the behavioral task began 10 d following surgery. The procedure was performed in strict accordance with local university regulations and the guidelines of the National Institutes of Health. Following injection, animals were maintained in a warmed cage under inspection for at least 2 h for recovery and then placed in a designated area in the animal facility.

Animals were divided into the following three groups: (1) rats not injected with virus; (2) rats injected with a control GFP virus; and (3) rats injected with a Gluk2 virus. Each of these groups will then be further divided into the following three behavioral groups: naive, trained, and pseudotrained (Table 1). Every cage contained one rat. Rats were kept under 12 h light/dark cycle conditions.

**Table 1:** Summary of the nine experimental groups

Naive-control	Naive-GFP only	Naive-GluK2
Trained-control	Trained-GFP only	Trained-GluK2
Pseudo-control	Pseudo-GFP only	Pseudo-GluK2

#### Virus preparation

We used the HIV-1 Lentiviral Vector System (Ramezani and Hawley, 2002) and a pUbiquitin-IRES-GFP plasmid (based on FU-mKate2-mem-Cer-SV2a-Wm2 13.024 kb). We exchanged the pUbiquitin promoter with CaMK2a promoter (thus allowing the virus to infect only the excitatory pyramidal neurons) and inserted the cDNA of GLU_R6_. Our final virus had the following composition:

5´CaMKIIaGluK2IRESGFP3´

#### Recovery from virus injection

As noted above, virus injection was followed by a 10 d resting period, after which olfactory discrimination training was performed in the complex OD task until rule learning was obtained. Notably, it has been shown that kainite-induced epileptic activity (for review, see Louboutin and Strayer, 2013). Specifically, GluK2 overexpression has been shown to produce seizures (Telfeian et al., 2000). On the other hand, it has been proposed that ketogenic diet-induced upregulation of GluK2 may prevent epileptogenesis by the inhibition of excitatory synaptic transmission at specific hippocampal pathways (Xu et al., 2008). To examine whether GluK2 overexpression results in epileptic events, rats were closely watched during the recovery period. None of the GluK2 virus-injected rats showed abnormal motor behavior.

#### Immunohistochemistry

Free-floating 50-mm-thick brain sections were collected in a cryostat (Leica) in PBS azide 0.05% and stored at 4°C until further use. Blocking was performed in 3% BSA with 0.3% Triton X-100 for 1–1.5 h at room temperature. Sections were incubated overnight with anti-GFP primary antibody at 4°C (1:1000 in PBS; anti-GFP IgG fraction; Thermo Fisher Scientific). Sections were then rinsed 4 times for 10 min each, with PBS incubated with the secondary antibody (1:2000 in PBS; goat anti-rabbit IgG; Thermo Fisher Scientific) for 2 h at room temperature. Sections were then washed twice for 10 min each and then were mounted on slides using (Fluoromount Aqueous Mounting Medium, Sigma-Aldrich).

## Results

### Synaptic stimulation induces long-term reduction of the postburst AHP in neurons from control rats only

To test whether complex OD learning and synaptically mediated AHP reduction share common pathways, we compared AHP reduction by tetanic synaptic stimulation in the piriform cortex of acute brain slices from animals trained in a complex olfactory discrimination paradigm and controls (naive or pseudotrained; see Materials and Methods; Fig. 1*A*). Trained rats learned how to discriminate between the two odors and chose the arm of the maze from which the rewarded odor originated. In the pseudotrained group, the reward was randomly assigned to one of the two odors in different trials. Naïve rats were not exposed to the maze or odors.

The postburst AHP in neurons from trained rats was not affected by a weak tetanus (20 pulses at 50 Hz). The averaged AHP before (7.9 ± 0.4 mV; *n* = 10) and after (7.8 ± 0.4 mV; *n* = 10) the repetitive stimuli was similar (*p* = 0.62, paired *t* test; Fig. 1*C*). In neurons taken from control rats, repetitive synaptic stimulation significantly reduced the AHP in piriform cortex pyramidal neurons (before stimulation, 9.7 ± 0.4 mV; after stimulation, 8.1 ± 0.4 mV; *n* = 12; *p* < 0.01, paired *t* test; Fig. 1*C*). Consequently, while the averaged AHP amplitude in neurons from trained rats was significantly lower than that in neurons from control rats (*p* < 0.01; Saar et al., 1998, 2001; Cohen-Matsliah et al., 2007; Chandra and Barkai, 2018) following tetanic synaptic stimulation, the difference between the averaged values was abolished (*p* = 0.64). Similar to what was previously reported in the hippocampus (Melyan et al., 2004), the tetanus-induced AHP reduction remained stable for the total recording period, up to 40 min after induction (Fig. 1*C*). The tetanus we used (20 stimuli at 50 Hz) is substantially milder than that required to elicit GluK2-dependent LTP (Petrovic et al., 2017).

### Kainate application reduces the AHP in neurons from control rats only

In hippocampus, the AHP reduction by tetanic stimulation is mediated by kainate receptors (Melyan et al., 2004). In piriform cortex of control rats, the application of 200 nm kainate significantly reduced the AHP (before stimulation, 9.6 ± 0.4 mV; after stimulation, 7.9 ± 0.6 mV; *n* = 10; *p* < 0.01, paired *t* test; Fig. 1*D*). This decrease outlasted the kainate washin and was stable for the total length of the recording (Fig. 1*D*).

In trained rats, after learning the complex olfactory discrimination paradigm, kainate application did not affect the AHP (before stimulation, 7.6 ± 0.7 mV; after stimulation, 7.8 ± 0.7 mV; *n* = 10; *p* = 0.29, paired *t* test; Fig. 1*D*). Thus, here too the averaged AHP amplitude in neurons from trained rats was significantly lower than in neurons from control rats (*p* < 0.05) before kainate application, and the difference between the averaged values was abolished following drug application (*p* = 0.84). We conclude that the AHP reduction resulting from complex olfactory discrimination learning mechanistically occludes AHP reduction both by tetanic stimulation and by nanomolar kainate application.

### Activity-induced AHP reduction is dependent on ERK and PKC activation

We then tested whether repetitive stimulation-induced, kainate-induced, and learning-induced long-lasting AHP reduction share common second-messenger systems. To this end, we performed three sets of complementary experiments.

**Figure 2.**
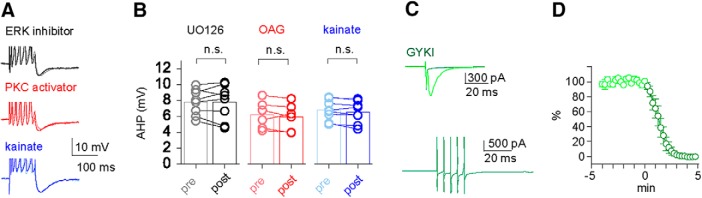
Activity-induced AHP reduction is mediated by intrinsic activity of second-messenger systems, and not by synaptic activation of kainate receptors. ***A***, Averaged typical traces in three neurons taken from naive rats before (light shade) and after (dark shade) repetitive synaptic stimulation. The AP peaks are clipped to facilitate comparison of the postburst AHP. Repetitive synaptic stimulation-induced post-AHP reduction is abolished by prior application of the ERK inhibitor UO126 (top traces), prior application of the PKC activator OAG (middle traces), or by prior kainate application (bottom traces). ***B***, Direct comparison of the absolute AHP amplitudes before and 20 min after repetitive synaptic stimulation in the presence UO126 (black), OAG (red), or kainate (blue). Each of the three treatments blocked the synaptic activity-induced AHP reduction. Note that the agonists kainate and OAG reduced the averaged AHP in naive neurons to the averaged value observed in neurons from trained rats, while the antagonist OU126 had no direct effect on the AHP. Values represent the mean ± SEM. ***C***, Synaptic currents evoked by stimulating the intrinsic fibers pathway (layer Ib) do not contain a kainate receptor-mediated component. The application of GYKI53655 completely blocked the synaptic currents (top trace). The bottom trace shows the lack of response to a train of five stimuli at 200 Hz in the same cells after GYKI53655 application. ***D***, Time line of the synaptic current amplitude before and after GYKI53655 application. Recordings were obtained in six pyramidal cells taken from three mice. Notably, the synaptic current amplitude is abolished in all recorded neurons. Values represent the mean ± SEM.

In the first experiment, we activated PKC. We previously showed that such persistent PKC activation is required for the long-term maintenance of AHP reduction (Seroussi et al., 2002; Cohen-Matsliah et al., 2007). We examined whether such persistent PKC phosphorylation, that results in long-term AHP reduction, prevents further AHP reduction by repetitive synaptic activation. As previously reported, in the presence of the PKC activator OAG, the amplitude of the AHP in neurons from naive rats is significantly lower than that recorded in control conditions, and resembles that observed in neurons from trained rats (Seroussi et al., 2002). In these conditions, tetanic stimulation did not further reduce the AHP in pyramidal neurons. The averaged AHP value measured in six neurons was 6.16 ± 0.7 before stimulation and 5.90 ± 0.7 after stimulation, indicating that once the AHP is reduced by PKC activation, repetitive synaptic stimulation has no further effect on its amplitude (Fig. 2*A*,*B*).

In the second experiment, we blocked ERK activation to examine whether interrupting the phosphorylation sequence that is required for long-term maintenance learning-induced AHP reduction (Cohen-Matsliah et al., 2007) would also prevent activity-dependent AHP reduction. Indeed, in the presence of the ERK inhibitor UO126, repetitive synaptic stimulation did not induce any reduction in the AHP amplitude. The averaged AHP value measured in eight neurons was 7.78 ± 0.6 before stimulation and 7.76 ± 0.8 after stimulation (Fig. 2*A*,*B*).

In the third experiment, we examined the effect of repetitive stimulation on the AHP after kainate application. The averaged AHP value measured in eight neurons was 6.76 ± 0.5 before stimulation and 6.52 ± 0.5 after stimulation, indicating that kainate application occludes further AHP reduction by repetitive synaptic activation (Fig. 2*A*,*B*).

Notably, synaptic stimulation, both single as well as repetitive, did not evoke any measurable kainite receptor-mediated EPSCs (Fig. 2*C*,*D*).

Synaptic currents were evoked by stimulation in the presence of the NMDA receptor blocker, d-AP5 (50 µm) and the GABA_A_ receptor blocker gabazine (1 µm). The synaptic current evoked in this condition was completely abolished by the application of tGYKI53655 (100 µm), a selective AMPA receptor antagonist (Fig. 2*C*,*D*). This concentration was chosen based on the original description of synaptically activated kainite receptors on CA3 pyramidal neurons in brain slices (Castillo et al, 1997; Vignes and Collingridge, 1997). There, the authors used 100 μm GYKI and accepted a partial block of kainite receptors, but yielded a full antagonism of AMPA receptors. We submit that, although we partially antagonize kainite receptors, we would still be able detect a significant expression of these receptors if expressed/anchored at synapses. Thus, synaptic currents evoked in this pathway are not mediated by kainate receptors. The downstream effects of kainate receptor activation are therefore not ionotropic but metabotropic.

### GluK2 mediates excitability change and complex OD learning

**Figure 3.**
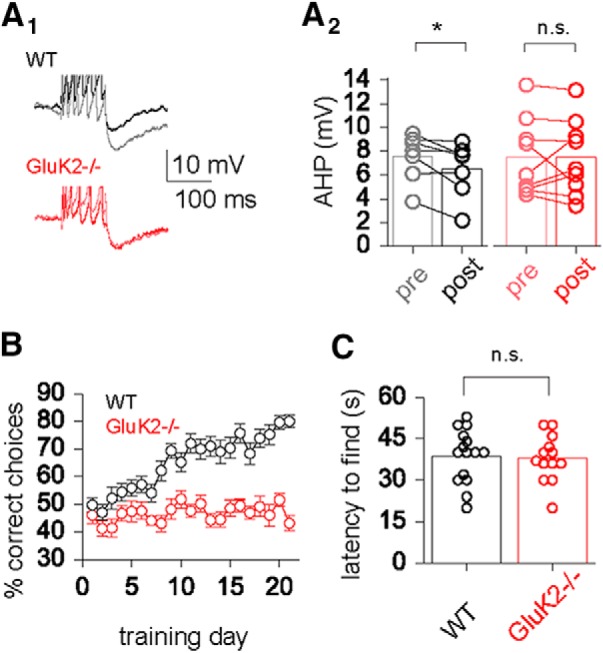
GluK2 activation is mandatory for AHP reduction and complex olfactory learning. ***A_1_***, Averaged typical traces of a neuron from GluK2^−/−^ mice (red) and wild-type littermates (black) before (light shade) and after (dark shade) kainate application. The AP peaks are clipped to facilitate the comparison of the postburst AHP. ***A_2_***, Direct comparison of the absolute AHP amplitudes before and 20 min after kainate application for GluK2^−/−^ mice (red) and wild-type littermates (black). ***B***, While wild-type mice (black) show a gradual learning curve, resulting in acquisition of the complex OD task for most (22 of 23) of the subjects, GluK2^−/−^ mice (red) performance mostly remains at the level of chance. Only 2 of 23 knock-out mice were able to learn the task. Average performance for each day was calculated for all 20 trials of the day. ***C***, Swarmplot of the latency to find the cookie in the buried food task in wild types (black) and GluK2^−/−^ mice (red). In this simple olfactory task, there is no difference between genotypes. Values represent the mean ± SEM. ^*^p < 0.05.

In the hippocampus, the activity-induced AHP reduction is GluK2 dependent (Fisahn et al., 2005). We therefore compared the effect of nanomolar kainate on piriform cortex layer II pyramidal neurons from wild-type mice and GluK2 knockouts. In neurons from wild types, kainate application (200 nm) reduced the AHP amplitude from 7.5 ± 0.8 to 6.5 ± 0.9 mV (*n* = 7; *p* < 0.05, paired *t* test; Fig. 3*A*). In contrast, kainate did not have an effect on the AHP in neurons from GluK2 knockouts (before stimulation, 7.6 ± 0.7 mV; after stimulation, 7.8 ± 0.7 mV; *n* = 10; *p* = 0.29, paired *t* test; Fig. 3*A*). Together, these data strongly indicate that GluK2 activation induces the long-term learning-induced AHP reduction, a strong cellular correlate of enhancement in learning capabilities (Saar et al., 1998; Zelcer et al., 2006; Chandra and Barkai, 2018).

Next, we examined the effect of GluK2 knockout on complex olfactory learning. Although the complex olfactory discrimination task was initially developed and used for training rats, wild-type mice are also capable of learning the task, but they require more time to reach the criteria for successful completion of complex OD learning (Fig. 3*B*). In sharp contrast, GluK2 knock-out mice are essentially incapable of completing the complex learning task (Fig. 3*B*).

To ensure that the GluK2 knock-out mice did not differ from the wild-type mice in general olfactory ability or motivational state, we performed a buried food test (also called cookie test; Yang and Crawley, 2009). In this paradigm, an appetitive and familiar food reward (butter cookie) is buried in the bedding of a clean cage. Animals are compared with respect to their ability to find the buried food and with the corresponding latencies. GluK2 knockouts did not differ from wild-type mice in the time they spent exploring the new environment until they found the cookie they had been previously habituated to (latency to find cookie: wild types, 38.7 ± 2.2 s, *n* = 15; GluK2^−/−^ mice, 37.9 ± 2.9 s; *n* = 13; *p* = 0.82; Fig. 3*C*), indicating that their decreased performance in the olfactory discrimination learning task cannot be attributed to a general olfactory impairment or to a lack of motivation.

**Figure 4.**
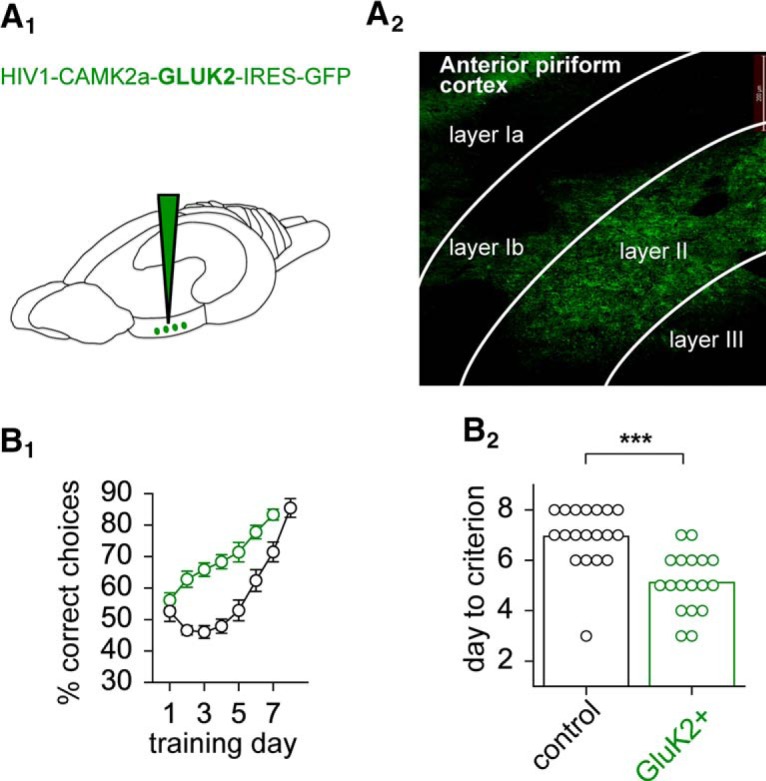
Enhanced complex olfactory learning is enhanced by GluK2 overexpression. ***A_1_***, Schematic of virus injection in the rat piriform cortex. ***A_2_***, Virus-infected pyramidal neurons in layer II indicated by GFP expression. Signal of GFP was enhanced using anti-GFP antibody. ***B_1_***, Time line of performance as a function of training day for GluK2-overexpressing rats (green) and controls injected with GFP virus only (black). To allow a more detailed look at the progress in performance for each day, the averaged percentage of correct choices for all 20 trials of the day are plotted. ***B_2_***, Swarmplot of the days needed to reach the criterion defined as a success rate of >80% in the last 10 trials. GluK2-injected rats (green) complete complex olfactory discrimination learning significantly faster than the control rats (black). Values represent the mean ± SEM. ^***^p < 0.001.

Finally, we overexpressed GluK2 in piriform cortex layer II pyramidal neurons (Fig. 4*A*). Our prediction was that GluK2 overexpression will considerably enhance complex learning in the olfactory maze. Indeed, piriform cortex GluK2 injected rats (*n* = 19) showed a considerably faster learning curve, compared with control rats (*n* = 18; Fig. 4*B*). Notably, the learning curves of the two groups began to separate on the second day of training (Fig. 4*B1*). Rats with piriform cortex overexpression of GluK2 reached the criterion for complex olfactory discrimination learning completion, 80% successes in the last 10 trials of the day, which is significantly faster than controls injected with GFP only (GluK2-injected group: 5.1 ± 0.3 training days, *n* = 19; GFP only-injected control group: 6.9 ± 0.3 training days, *n* = 18; *p* < 0.001; Fig. 4*B2*). These genetic modifications suggest that GluK2 receptors have a central role in complex olfactory learning.

## Discussion

The first physiologic modification that is detected in single neurons during and after complex olfactory learning is enhanced intrinsic neuronal excitability by reduction of the slow AHP (Saar et al., 1998, 2002, 2012). We demonstrate in the piriform cortex that the synaptically activated GluK2-type glutamate receptor is the upstream mediator of this AHP reduction. Genetic manipulations (transgenic knock-out and region-specific virally mediated overexpression) demonstrate the necessity and sufficiency of GluK2-mediated intrinsic excitability changes for complex OD learning. We therefore provide a direct link between metaplasticity at the cellular level and complex learning at the behavioral level.

### Noncanonical signaling of GluK2 receptors and learning

Previous studies in the hippocampus suggest that glutamatergic synaptic transmission reduces the AHP by noncanonical signaling of the GluK2 subtype glutamate receptor (Melyan et al., 2002, 2004; Fisahn et al., 2005; Grabauskas et al., 2007; Melyan and Wheal, 2011). In the piriform cortex, we provide a direct link between this cellular mechanism and complex olfactory learning. Complex olfactory learning occludes *in vitro* stimulation paradigms that result in AHP reduction in neurons from control animals (Fig. 1). Genetic knockout of GluK2 abolishes kainate-mediated reduction of the AHP and complex olfactory learning (Fig. 3). In addition, region-specific overexpression of GluK2 receptors enhances complex olfactory learning performance (Fig. 4). While noncanonical signaling of ionotropic glutamate receptors has been described, its functional relevance on a behavioral level is unclear (Valbuena and Lerma, 2016). This is the first direct demonstration that noncanonical signaling of ionotropic glutamate receptors has a selective impact on higher cognitive abilities.

### Long-lasting AHP reduction is induced and maintained via metabotropic activation

Several studies indicate that the learning-induced reduction in neuronal adaptation and in AHP amplitude result from reduction in an acetylcholine-sensitive Ca^2+^-dependent potassium current (Sanchez-Andres and Alkon, 1991; Saar et al., 2001; Power et al., 2002). In particular, learning-induced enhanced excitability is mediated by long-term reduction in the conductance of the sI_AHP_, in both the piriform cortex (Brosh et al., 2006) and hippocampus (Power et al., 2002). Olfactory discrimination learning-induced AHP reduction in the piriform is also PKC dependent (Seroussi et al., 2002) and ERK dependent (Cohen-Matsliah et al., 2007). Our data show that the same second-messenger systems are also required for activity-induced induction of the long-lasting AHP reduction. Moreover, synaptically induced and kainate-induced AHP reduction is occluded by prior complex learning; *in vitro* AHP reduction abolishes the difference in the average AHP amplitude between neurons from trained and control animals. Thus, our data support the hypothesis that glutamate secretion, which occurs during learning, induces long-lasting AHP reduction via a metabotropic mechanism. It should be noted that a possibility for AHP “contamination” by GABA_B_ receptor-mediated synaptic inhibition cannot be completely ruled out. However, such a scenario is highly unlikely; OD learning-induced postburst AHP reduction is mediated by a decline in the conductance of the current that mediated the AHP (Saar et al., 2001), whereas synaptic inhibition is enhanced after learning (Kfir et al., 2014). Moreover, intracellular application of the calcium chelator abolished the differences in the AHP amplitude between neurons from trained animals and controls (Saar et al., 2001). This evidence implies that the OD learning-induced AHP reduction is induced and maintained by modulation of intrinsic membrane currents.

### Functional impact of global increases in intrinsic excitability

Widely spread AHP reduction, and the resulting enhancement of neuronal excitability, is an ideal candidate for preparing neuronal networks for subsequent learning (Chandra and Barkai, 2018). This global unspecific upregulation of the sensitivity for encoding content has to be distinguished from the synapse-specific encoding of content. Several lines of evidence support the notion that an increase in intrinsic excitability triggered by the GluK2-mediated AHP reduction is an ideal cellular mechanism for complex learning; one prerequisite for a metaplastic mechanism that primes a neuronal network for learning is that the metaplastic state change needs to last longer than its induction. This temporal delay criterion is met by GluK2-mediated AHP reduction. Our *in vitro* experiments as well as the aforementioned studies in the hippocampus demonstrate that the GluK2-mediated AHP reduction is long lasting. This is in contrast to the previously reported neuromodulatory AHP reduction mediated, for example, by noradrenergic stimulation of β1 receptors (Madison and Nicoll, 1986). Importantly, learning-induced postburst AHP reduction in the piriform cortex is the first cellular modulation that is detected after complex OD learning; it is induced 2 d before enhanced synaptic connectivity appears (Saar et al., 1998, 2012; Knafo et al., 2005; Ghosh et al., 2015).

Changes in intrinsic excitability have been linked to plasticity and learning before. During auditory fear conditioning, neurons with enhanced intrinsic excitability are preferably recruited into new memory traces (Gouty-Colomer et al., 2016). Moreover, activity-induced synaptic strengthening also occurs in more excitable neurons. LTP is more readily induced when the AHP is reduced (Sah and Bekkers, 1996; Norris et al., 1998). Also, following complex olfactory discrimination learning the postburst AHP is reduced and neuronal excitability is transiently enhanced in CA1 pyramidal neurons for 3 d. During this period, the rats show enhanced learning capability in a different hippocampus-dependent task, the Morris water maze (Zelcer et al., 2006).

### Complex olfactory discrimination learning and rule learning

Previously, we demonstrated complex olfactory learning results in the general enhancement of learning capabilities and thus can be described as rule learning (“learning how to learn,” or “rule learning”). By acquiring an abstract set of rules when they reach the training criterion the first time, the rats reach the criterion much faster when presented with a different set of odors in the same task. Long-lasting AHP reduction in the pifirorm cortex is a prerequisite for rule learning (Saar et al., 1998; Zelcer et al., 2006; Chandra and Barkai, 2018). While we did not demonstrate the involvement of GLUK2 in rule learning, it is tempting to speculate that the GLUK2-mediated reduction of the AHP during complex OD learning is the mechanism underlying the AHP reduction necessary for rule learning.

To conclude, our data suggest that GluK2-mediated AHP reduction, and the subsequent enhancement in neuronal excitability within the piriform cortex, is the mechanism that enables neuronal ensembles to enter into a state of metaplasticity at the cell and network levels that constitutes the mechanistic basis of complex OD learning. It will be of interest whether other brain areas, such as for example the hippocampus, are not only equally involved in this kind of complex learning, but also use similar molecular and cellular mechanisms. Interestingly, aberrant metaplasticity has been linked to cognitive dysfunction (Hulme et al., 2013). Indeed, GluK2 loss in humans results in intellectual disability (Motazacker et al., 2007). Our study is the first to provide a mechanistic hypothesis of how the loss of GluK2 in humans can result in intellectual disabilities by impairment of the AHP-related metaplasticity underlying complex learning and eventually rule learning. Modulation of this specific pathway suggests new avenues for the treatment of cognitive dysfunction as well as cognitive enhancement.

## Synthesis

Reviewing Editor: Upinder S. Bhalla, National Centre for Biological Sciences, Tata Institute of Fundamental Research; Decisions are customarily a result of the Reviewing Editor and the peer reviewers coming together and discussing their recommendations until a consensus is reached. When revisions are invited, a fact-based synthesis statement explaining their decision and outlining what is needed to prepare a revision will be listed below. The following reviewer(s) agreed to reveal their identity: NONE.

The authors have substantially improved the manuscript, and have removed the

key point of concern of the reviewers. There are still some points that

they must address, but these can be done quickly without further experiments:

1. The authors must better specify the details of the odors used: their

chemical composition, whether single or mixtures, sources, and concentrations.

2. The authors must discuss the contribution of non-intrinsic sources to the

slow hyperpolarization.

3. The authors should discuss the implications of partial block of Kainate.

Minor point:

The statement “necessary and sufficient” (line 30 in the abstract) is too

strong. OD learning is a complex network task and one cannot put it all down

to Kainate receptors. Also line 39 and 387.

The detailed comments of the reviewer are attached.

----------------------------------------------------------

The authors have addressed most of my concerns. Overall the discussion is more careful and more in line with the data they provide. I have two minor questions left:

Odors used: in the methods, the authors have added the following sentence: “Odors used in this study were: apple, skin musk, lavender ”, stating that these are commercial odors that are regularly used in the cosmetics and food industry. Are they pure chemically, or mixtures synthesized from many compounds? Were they chemicals that were purchased and if so, from which companies were they from?

Regarding the Minor point (1): My original concern was that the slow hyperpolarization evoked in the cell after depolarization may not be entirely due to intrinsic conductances. In the study that the authors cite (Brosch et al) as evidence that the hyperpolarization is a sAHP, an application of apamin, an SK channel blocker, seems to only partially reduce the hyperpolarization. That is, the hyperpolarization following the burst firing is not just from SK channels. Due to the recurrent circuitry (see Franks et al., DOI: https://doi.org/10.1016/j.neuron.2011.08.020), strong depolarizations, even of a single cell, is likely to recruit local inhibitory neurons. If the authors are unable to determine the cellular mechanism underlying the hyperpolarization that follows burst firing, they need to at least acknowledge the potential contribution from non-intrinsic sources.

As a side note related to the above, the antagonist that the authors used (in the new data presented in Figure 2) is expected to block kainate receptor partially at the dose they used (IC50 for GluK3 homomeric receptor is 63 uM and at GluK2b/GluK3 heteroreceptos is 32 uM; the dose the authors used is 100 uM, higher than these IC50's), so this does not provide the strongest evidence for a lack of kainate receptor in the synaptic transmission (also note that the authors' analysis on the kainate contribution was on recurrent excitation only, not excitatory transmission to inhibitory neurons).

## Author Response

Synthesis of Reviews:

Computational Neuroscience Model Code Accessibility Comments for Author (Required):

N/A

Significance Statement Comments for Author (Required):

N/A

Comments on the Visual Abstract for Author (Required):

N/A

Synthesis Statement for Author (Required):

Synthesis (Summary of reviews):

The reviewers felt that the study was potentially of interest, but was not

able to demonstrate its findings unambiguously. Specific points of concern

were that:

1. The paper lacked a clear definition and demonstration of meta-learning.

This requires a clear dissociation of meta-learning from other forms of

learning.

2. The presentation of data is not always sufficient, particularly with

identification of mechanisms and signaling pathways. Together the presented data do not unambiguously show meta-learning.

3. There was confusion in the use of SD and SE for the data in text and figures.

In addition there were several specific points that the reviewers brought up

that should be addressed. The reviewers felt that addressing these points and

the points above would require substantial work including additional

experiments.

1. According to the reviewers' suggestion, we omitted the term meta-learning from the manuscript. The papers in now entitled “A cellular mechanism underlying enhanced capability for complex olfactory discrimination learning”. The text throughout the manuscript was modified accordingly and refers now to the behavioral phenomenon of complex olfactory discrimination learning.

2. We added a new figure (figure 2), in which we describe the effect of the PKC and ERK second messenger systems on activity-induced AHP reduction and show that the synaptic response is not mediated by ionotropic kainate receptors.

3. We now use SD throughout the manuscript and figures.

All other comments are address point by point below.

-

Reviewer 1:

1. There is no clear definition and explanation of meta-learning. As a neuroscience term, first proposed by Doya, it describes how neuromodulators (NE, Ach, 5-HT, dopamine etc) facilitate distributed learning mechanisms. In this work, the authors equal meta-learning to rule learning and attribute it to a synaptic glutamate effect. If it's a synaptic mediated effect, what could account for the global increase in excitability (not input-specific as in synaptic plasticity)? Why would odor-specific training differ from pseudo-random odor training in altering cell excitability since both cohorts receive the same inputs (two odors and water reward)?

We fully accept this comment. We now modified the manuscript to refer only to complex olfactory discrimination (OD) learning and not to meta-learning.

The difference in intrinsic excitability in neurons from trained and pseudo trained rats has been documented consistently by others and us in the two last decades. Such long-term enhancement of neuronal excitability can be induced in-vivo only during the learning process. Exposure to the odors and maze by itself does not produce this effect (described in Saar et al., 1998, 2001, Saar and Barkai 2003; Zelcer et al., 2006). However, such modification in the intrinsic neuronal excitability can be induced in-vitro under conditions of high frequency stimulation that evokes am EPSP with a significant amplitude (10 mV in our study). In this work we show that:

1. Synaptically evoked AHP reduction can be induced in neurons from controls but not form trained rats. Moreover, synaptic activation reduces the post burst AHP in neurons from control rats to the same value recorded in neurons from trained rats. The same is true for direct kainate application on the recorded neurons.

2. Synaptic activation fails to reduce the AHP in neurons from GluK2 knockout mice. In parallel, these mice are incapable of complex OD learning.

3. Virus-induced GluK2 overexpression in piriform cortex pyramidal neurons enhances complex OD learning considerably.

We conclude that taken together these findings support the notion that GluK2 has a key role in complex OD learning. Hopefully the revised manuscript presents our claim more clearly.

2. The main point that the authors propose is that GluK2 mediates meta-learning through altering cell excitability globally. However, there is no demonstration of meta-learning in this study. The authors used a complex odor learning (a single learning) but no demonstration of the meta-learning effect (i.e. this learning facilitates a future learning with similar form). This makes it weak to correlate learning induced changes with the production of meta-learning.

As noted is our response to comment #1, the manuscript is now modified and refers only to complex OD learning. It is worthwhile noting that we showed in a previous study that complex olfactory-discrimination learning results in a general enhancement of learning capabilities, for example in the Morris water maze (Zelcer et al., 2006) .

3. Fig.1, The authors could first show the complex learning can serve as a rule-learning to facilitate a later learning (e.g. learning with a different odor pairs), in parallel, this complex learning excludes synaptic stimulation and Kainate induced AHP reduction. Additionally, it's not shown whether the synaptic stimulation protocol used activates GluK2 receptors and how this protocol is chosen to mimic a learning effect. In fact, the authors pointed out this protocol (20@50Hz) is substantially milder than required to elicit GluK2-dependent LTP. Would it be more practical to use a LTP protocol that resembles learning?

We should stress that, as the reviewer rightfully asks, we have shown in our previous publications that complex olfactory discrimination learning can serve as a rule-learning to facilitate a later learning (e.g. learning with a different odor pairs). Some of these papers are quoted here (Saar et al., 1998, 2001; Saar and Barkai 2003). In these studies, we showed repeatedly that ones the rats acquire the rule they learn to discriminate between new pairs of odors in one or two days of training.

To fully clarify this point, we added the following paragraph at the end of the discussion (page 20, line 13):

“Complex olfactory discrimination learning and rule learning

Previously, we demonstrated complex olfactory learning results in the general enhancement of learning capabilities and thus can be described as rule-learning (‘learning how to learn‘, or ‘rule learning’). By acquiring an abstract set of rules when they reach the training criterion the first time, the rats reach criterion much faster when presented with a different set of odors in the same task. Long-lasting AHP reduction in the pifirorm cortex is a prerequisite for rule learning (Saar et al., 1998, Zelcer et al., 2006, Chandra and Barkai 2017). While we did not demonstrate the involvement of GLUK2 in rule learning, it is tempting to speculate that the GLUK2 mediated reduction of the AHP during complex OD learning is the mechanism underlying the AHP reduction necessary for rule learning.”

Regarding the parameters for synaptic repetitive synaptic stimulation, our previous studies led us to suggest that enhanced intrinsic neuronal excitability, such that is expressed in most excitatory neurons in the relevant neuronal network, is the cellular mechanism underlying complex OD learning (rule learning) (for example, Saar et al.1998, 2001; Zelcer et al. 2006; Motanis et al., 2014. These data are reviewed recently in Chandra and Barkai 2017). We thus preferred to use a repetitive synaptic stimulation that is sufficient to induce long-term reduction of the post burst AHP, similar to the effect obtained by kainate application, but is considered too weak to induce LTP.

We fully accept the comment regarding synaptic activation by kainate. We added a new set of data and figure, showing that the effect is not mediated by ionotropic kainate receptors as follows (page 15, line 16):

“Notably, synaptic stimulation - both single as well as repetitive - did not evoke any measurable kainate-receptor mediated excitatory postsynaptic currents. (Figure 2 C, D). Synaptic currents were evoked by stimulating in the presence of the NMDA-receptor blocker, D-AP5 (50uM) and the GABAA-receptor blocker, Gabazine (1uM). The synaptic current evokes in this conditions was completely abolished by application of the GYKI (100uM), the AMPA-receptor antagonist. Thus, synaptic currents evoked in this pathway are not mediated by kainate receptors.”

These data shown in the new figure 2 (C, D).

4. Fig.2, Here, the authors demonstrated that GluK2 knockout prevents the initial complex learning, there is no evidence for meta-learning (the influence on a future learning). Additionally, while it's not ideal to use different species in the same study, it could be improved by providing some overlapping characterizations (e.g. repeat some of the figure 1 experiments in mice).

As noted above, the term meta-learning is now omitted form the manuscript. Our study combines experiments in mice and rats since all the previous work on complex OD learning-induced enhancement of intrinsic excitability was performed in rats (examples are noted in our response to the previous comments). In the present work we added experiments performed in in mice since we aimed to show causal relations between Gluk2 activation, AHP reduction and complex OD learning. To that end we used GluK2 knockout mice.

4. Fig.3, again, no demonstration of meta-learning effect. I think an additional non-infused control group that demonstrates facilitated learning following the initial complex learning would be good. In this case, the 2nd learning would be facilitated (presumably via GluK2 activation and AHP reduction), similar to the GluK2 overexpression case.

As noted above (response to comment 2), We demonstrated in quite few of our previous publications that such complex OD learning facilitates subsequent learning, both in the same olfactory discrimination task (Saar et al., 1998, 2001; Saar and Barkai 2003) and in a spatial learning task, the Morris water maze (Zelcer et al. 2006). As noted above, we accept the reviewer's comment regarding the use of the term meta-learning. It is now replaced with complex OD learning.

Minor points

1. Positive and negative odors used, concentrations and whether they were randomized in the maze arms should be described.

We have been working with commercial odors that are regularly used in the cosmetics and food industry. We added the odors used in this study to the methods section: “Odors used in this study were: apple, skin musk, lavender”. According to the training paradigm, each odor may be applied to each of the arms. In each trial one arm is selected randomly as for the correct odor and second arm for the wrong odor. Thus for each trial the two arms are chosen randomly, so no odor is localized to a specific arm.

2. Why the data are presented as mean {plus minus}SD in the text, but mean {plus minus}SE in the figures?

All data is now represented as mean+SEM

Reviewer 2:

1. The most fundamental problem is that the study does not specifically address meta-learning. There is no attempt to dissociate meta-learning process from learning-related changes. For example, in two cases (data presented in Figures 2 &3; GluK2 knockout mice and rats injected with GluK2-expressing viruses respectively), animals learn at different rates from control cases. However, it is unclear if these manipulations have any relation to meta-learning, as they do not present how these animals learn new odors pairs subsequently.

As noted in our response to comment #3, the manuscript is now modified and refers to complex OD learning rather to meta-learning. Our reference to the term ‘rule-learning’ relies on our previous findings, were we showed repetitively that once the animals learn the complex task, they would discriminate between the second pair of odor within one-two training days. These results are described in detail in several paper cited in the manuscript (see Saar et al, 1998, 2001). Moreover, they would also excel in other unrelated task that demand complex learning capabilities (Zelecer et al., 2006). Having said that, according to reviewers' suggestion, we are now use mostly the term “complex olfactory discrimination learning”.

2. In Results, the authors describe at length about their result regarding signaling pathways known to be involved in regulating sAHP. However, they present no figures associated with this section. It is difficult to assess the validity of this result without data/figure.

We accept the reviewer's suggestion. The new figure 2 in the revised manuscript describes the results of experiments aimed to reveal the signaling pathway convention between kainate, as well as the ERK and PKC second messenger systems activation, and slow

AHP reduction. In this figure we also present new data describing results of experiments requested by reviewer #1.

3. While their title claims that this is a ‘novel’ cellular mechanism, it seems that a number of (their) previous works have analyzed the reduction in sAHP associated with (meta-) learning. Together with the above points, a more appropriate title seems to be something like “pathways involved in the reduction of sAHP associated with olfactory discrimination learning”.

According to the reviewers' comments, we now modified the title to “A cellular mechanism underlying enhanced capability for complex olfactory discrimination learning”

Minor points

1. It is unclear what cellular mechanism underlies the hyperpolarization that accompanies the burst firing. While the authors suggest that this is sAHP - i.e., the origin of hyperpolarization is intrinsic to the cell being recorded - given the recurrent circuitry of the piriform cortex, it is difficult to rule out a contribution from recurrent inhibition. The authors should block GABAergic transmission to dissect the underlying mechanism.

We should note that the slow AHP is not evoked by synaptic stimulation, but by injecting current via the recording electrode, thereby evoking only intrinsic currents. We showed in our previous studies that slow AHP is reduced in by reduction of an intrinsic calcium-dependent potassium current (Saar et al., 2001,). In a later study (Brosh et al., 2006), this current was identified as the sIAHP current. Our previous studies show that synaptic inhibition in actually enhanced after learning (Ghosh et al., 2015 and several other papers that are not cited here). The piriform cortex is very prone to epilepsy. Blocking GABAergic transmission unavoidably results in wide epileptic activity within the slice, with strong spontaneous synaptic currents in the recorded neurons. In these conditions, it is not possible to distinguish between intrinsic and synaptic potentials in the recorded neuron.

2. Regarding the induction of long-term reduction in sAHP with kainate application, from the example waveforms, it seems that many aspects are different from that induced by synaptic stimulations. The biggest difference seems to be the time course of the hyperpolarization (not to mention the magnitude of fAHP). It is unclear if the authors are looking at the same phenomenon.

We argue that slow AHP reduction by kainate application and repetitive synaptic stimulation are induced via the same mechanism since AHP reduction with one treatment occludes the same effect with the other treatment. These data are described in the results (Page 15, line 12): “ In the third experiment, we examined the effect of repetitive stimulation on the AHP after kainate application. The averaged AHP value measured in 8 neurons was 6.76+0.5 mV before stimulation and 6.52+0.5 mV after stimulation, indicating that kainate application occludes further AHP reduction by repetitive synaptic activation.”

These results are also shown in the new figure 2.

3. Was the virus injection unilateral or bilateral? What coordinates were used for injection?

The virus was injected bilaterally. The coordinates in the piriform cortex area were:

AP: + 1.3, ML: {plus minus} 3.5, DV: -8.

This is now added to the Methods section.

4. In Methods, they describe that they used 0.2 ml water reward per trial. This is a large volume, especially for mice. Using such large volumes is likely to substantially interfere with the motivation level, which is critical since the authors use data from the last 10 trials (in a 20-trial session) for analysis. For clarity, the authors should illustrate how animals perform over an entire session.

Behavioral data for mice training is indeed presented for all the 20 trials of the day. This is now noted in the figure legend (figure 3B): “ Average performance for each day was calculated for all 20 trials of the day.”

5. It is unclear what exactly is being tested with a cookie-test. Clearly it is an easier task, but it is unclear if it is a task where animals improve (getting faster at finding the cookie). A lack of difference in the initial behavioral performance is less convincing.

The cookie test was conducted solely as a control, to rule out olfactory, motor or motivational deficits in the GluK2 knockout mice. As the reviewer correctly comments, there is indeed no learning involved in this task. Since the cookie was buried in random positions in the cage, the animals have no opportunity to improve their performance.

The corresponding text in the results has been changed to clarify this, and now reads (page 16, line 15):

“To ensure that the GluK2 knockout mice did not differ from the wild type mice in general olfactory ability or motivational state, we performed a buried food test (also called cookie test, Yang and Crawly, 2009). In this paradigm, an appetitive and familiar food reward (butter cookie) is buried in the bedding of a clean cage. Animals are compared with respect to their ability to find the buried food and with the corresponding latencies. GluK2 knockouts did not differ from wild type mice in the time they spent exploring the new environment until the found the cookie they had been previously habituated to (latency to find cookie: wild types=38.7{plus minus}2.2 s, n=15, GluK2-/-=37.9{plus minus}2.9 s, n=13, p=0.82, Fig. 2C), indicating that their decreased performance in the olfactory discrimination learning task cannot be attributed to a general olfactory impairment or to a lack of motivation.”

6. The authors describe that they used commercial odors that are regularly used in the cosmetics and food industries for the behavioral training. They should state which odors are used for which groups of animals. As the strength of different odors varies, this is crucial information to assess the behavioral data presented in this study.

The odors used in these study were; apple, skin musk, lavender. This is now noted in the methods. We showed in our previous studies that animals do not show preference to a particular odor, since before they learn the task they enter the correct arm about 50% of the time. In addition, the pseudo trained animals chose the “correct odor” about 50% of the time. These data are shown in our previous studies (see Saar et al., 1998 and Saar et al., 2001, for example). These results are repeated in the present study, as can be seen in figures 3B and 4B1.

7. Statistics Largely OK - but they use SD and SEM for the same data, but differently for text and figures, which they should not do. As a minor comment, there is no indication that the distributions are normal (they do not present such data in the figures).

All data is now represented as mean+SEM.
